# P2X7 receptor activation leads to NLRP3-independent IL-1β release by human macrophages

**DOI:** 10.1186/s12964-023-01356-1

**Published:** 2023-11-23

**Authors:** Judith Bockstiegel, Jonas Engelhardt, Günther Weindl

**Affiliations:** Pharmacology and Toxicology Section, Pharmaceutical Institute, University of Bonn, 53121 Bonn, Germany

**Keywords:** P2X7 receptor, IL-1beta, Human macrophages, Toll-like receptors, NLRP3 inflammasome

## Abstract

**Background:**

The purinergic receptor P2X7 plays a crucial role in infection, inflammation, and cell death. It is thought that P2X7 receptor stimulation triggers processing and release of the pro-inflammatory cytokine interleukin (IL)-1β by activation of the NLRP3 inflammasome; however, the underlying mechanisms remain poorly understood.

**Methods:**

Modulation of IL-1β secretion was studied in THP-1 macrophages. Adenosine 5’-triphosphate (ATP), BzATP, nigericin and pharmacological inhibitors of P2X receptors, inflammatory caspases and the nucleotide-binding oligomerization domain-like receptor family pyrin domain-containing protein 3 (NLRP3) inflammasome were used to characterize signaling.

**Results:**

In primed macrophages, IL-1β release was increased after P2X7 receptor activation by ATP and 2,3-O-(4-benzoylbenzoyl)-ATP (BzATP). Pharmacological inhibition or genetic knockout of NLRP3 does not completely inhibit IL-1β release in TLR2/1-primed macrophages. Increase in extracellular K^+^ as well as inhibition of caspase-1 or serine proteases maintained IL-1β release in macrophages stimulated with P2X7 receptor agonists at 50%.

**Conclusions:**

Our findings suggest a previously unrecognized mechanism of P2X7 receptor mediated IL-1β release and highlight the existence of an NLRP3-independent pathway in human macrophages.

Video Abstract

**Supplementary Information:**

The online version contains supplementary material available at 10.1186/s12964-023-01356-1.

## Background

IL-1β is a highly potent and proinflammatory cytokine [1]. While IL-1β facilitates antibacterial and antiviral effects, it is also strongly linked to inflammatory and autoimmune diseases [2–4]. IL-1β is released following a two-step process in macrophages. The first step, known as priming, is characterized by nuclear translocation of NF-κB which facilitates the expression of pro-IL-1β and NLRP3-associated proteins. The second step, known as activation, leads to NLRP3 inflammasome assembly and the processing of pro-caspase-1 into active caspase-1. Caspase-1, in turn, processes pro-IL-1β into its active form IL-1β [5, 6]. In human macrophages, priming can be initiated by multiple stimuli leading to activation of Toll-like receptors (TLR), Tumor necrosis factor (TNF) receptors, or IL-1 receptors [5, 7], while in human monocytes the priming step is dispensable [8–10]. Activation can be achieved by danger associated molecular patterns (DAMPs) such as ATP, particulate matter such as uric acid crystals, and pore-forming toxins such as nigericin. The common denominator connecting these NLRP3 activating stimuli is K^+^ efflux [11, 12]. However, the precise mechanisms by which the NLRP3 inflammasome responds to a variety of stimuli are poorly understood.

ATP is one of the best studied DAMPs [13, 14]. Extracellular ATP activates cell surface P2X and P2Y receptors. P2X receptors are membrane-bound ion channels that are permeable to calcium, sodium, and potassium [15, 16]. The P2X receptor most involved in inflammation is the P2X7 receptor, whose affinity for ATP is low. However, high ATP concentrations at sites of inflammation or tumors can lead to activation of the receptor [17, 18]. Activation of the P2X7 receptor by ATP or the more potent agonist BzATP [18, 19] serves as an activation signal that leads to IL-1β release mediated by K^+^ efflux and activation of the NLRP3 inflammasome [17].

To date, inflammatory signaling has often been studied in mouse bone marrow-derived macrophages [11], however, human macrophages have rarely been discussed. In this study, we investigate the underlying mechanism of P2X7 receptor mediated IL-1β release in human macrophages. Our findings are supported by using BzATP, which eliminates the possible confounding effects of high ATP concentrations. Our data suggest the existence of two signaling pathways leading to the release of IL-1β after activation of the P2X7 receptor, with both mechanisms being potentially NLRP3 independent in TLR-primed macrophages. One mechanism is dependent on K^+^ efflux and requires activation of caspase-1 and serine proteases, while the other mechanism is independent of K^+^ efflux, caspases, as well as serine, cysteine, and aspartic proteases.

## Materials and methods

### Cell culture

THP-1 cells (ACC 16, DSMZ-German Collection of Microorganisms and Cell Cultures GmbH, Braunschweig, Germany) or THP-1 KO NLRP3 cells (thp-konlrp3z, Invivogen, Toulouse, France) were cultured in RPMI 1640 (11530586, Fisher scientific, Schwerte, Germany) containing 100 U/ml penicillin, 100 μg/ml streptomycin (P4333), 2 mM L-glutamine (G7513, both from Sigma-Aldrich, Taufkirchen, Germany) and 10% heat-inactivated fetal bovine serum (FBS; S0615, Sigma-Aldrich, Taufkirchen, Germany) at a density of 2–8 × 10^5^ cells/ml. Cells were used from passage 4 to 25 and maintained at 37 °C in a humidified atmosphere of 5% CO_2_ and 95% air. Cell lines were regularly tested negative for mycoplasma contamination (VenorGeM Classic Mycoplasma PCR detection kit, 11–8100, Minerva Biolabs, Berlin, Germany). For generating THP-1 derived macrophages, THP-1 monocytes were seeded into 24-well plates at a density of 4 × 10^5^cells/ml in growth medium including 25 ng/ml PMA (phorbol 12-myristate 13-acetate; tlrl-pma, Invivogen, Toulouse, France). After 48 h, adherent cells were carefully washed with PBS (phosphate buffered saline; P04-53500, Pan Biotechne, Aidenbach, Germany) and rested in PMA-free medium for 24 h. PBMCs were isolated from buffy-coat donations (Institute of Experimental Haematology and Transfusion Medicine, University Hospital Bonn) by density gradient centrifugation using Bicoll separation media (BS L6115, Bio&Sell, Nuremberg, Germany). HEK-blue IL-1β cells were cultured in Dulbecco's modified Eagle's medium (DMEM, P04-03500, Pan Biotechne, Aidenbach, Germany) containing 4.5 g/l glucose, 10% (v/v) heat-inactivated fetal bovine serum (FBS; S0615), 2 mM l-glutamine (G7513), 100 U/ml penicillin, 100 μg/ml streptomycin (P4333, all from Sigma-Aldrich, Taufkirchen, Germany), 100 μg/ml normocin, 100 μg/ml zeocin (selective antibiotics, ant-nr-1, ant-zn-1, both from Invivogen, Tolouse, France). Cells were used from passage 3 to 20 and maintained at 37 °C in a humidified atmosphere of 5% CO_2_ and 95% air. To determine if THP-1 stimulated cells secrete bioactive form of IL-1β a total of 50 μl of THP-1 supernatant was transferred to a 96-well tissue culture–treated plate and mixed with 5 × 10^4^ IL-1β reporter cells in test media (culture media without normocin and zeocin) and incubated for 20 h at 37 °C. After IL-1β receptor stimulation of HEK-Blue cells, NF-κB and AP-1 activation-induced production of secreted embryonic alkaline phosphatase (SEAP) that can be determined with the colorimetric substrate QuantiBlue (rep-qbs, Invivogen, Toulouse, France) by reading the optical density (OD) at 620 nm.

### Cell stimulation

PBMCs as well as THP-1 monocytes and differentiated THP-1 derived macrophages were preincubated with LPS from *Escherichia coli* 0111:B4 (tlrl-3pelps), Pam_2_CSK_4_ (tlrl-pm2s-1), Pam_3_CSK_4_ (tlrl-pms-1, all from Invivogen, Toulouse, France) or growth medium for 3 h. Afterwards PBMCs and THP-1 macrophages were stimulated for additional 3 h or THP-1 monocytes for 6 h with 300 μM BzATP (2'/3'-O-(4-Benzoylbenzoyl)adenosine-5'-triphosphate, NU-1620–25), 5 mM ATP (Adenosine 5´-triphosphate, NU-1010-100G, both from Jena Bioscience, Jena, Germany) 10 μM nigericin (4312/10, Tocris Bioscience, Bristol, United Kingdom). In selected experiments, cells were preincubated with the non-competitive P2X7 receptor antagonist A804598 (1 μM, 4473, Tocris Bioscience, Bristol United Kingdom), irreversible P2X7 receptor antagonist oxidized ATP (oxATP, 300 μM, 505758, Merck, Darmstadt, Germany), P2X4 receptor antagonist 5-BDBD (25 μM, SML0450-5MG, Sigma-Aldrich, Taufkirchen, Germany), P2X receptor antagonist PPADS (100 μM, 0625, Tocris Bioscience, Bristol, United Kingdom), NLRP3 inhibitor MCC950 (10 μM, 5479, Tocris Bioscience, Bristol, United Kingdom) or Bay 11–7082 (20 μM, B5556, Sigma-Aldrich, Taufkirchen, Germany), Caspase-1 inhibitor Ac-YVAD-cmk (40 μM, 10014, Biomol, Hamburg, Germany), pan-caspase inhibitor Z-VAD-fmk (40 μM, tlrl-vad, Invivogen, Toulouse, France), serine protease inhibitor AEBSF (300 μM, 50985.100, Biomol, Hamburg, Germany), cysteine protease inhibitor E64 (10 μM, 324890, Merck, Darmstadt, Germany), aspartic protease inhibitor pepstatin A (50 μM, 2936, Carl Roth, Karlsruhe, Germany) 1 h before stimulation. To determine the optimal concentration of caspase inhibitors required to inhibit IL-1β secretion, we incubated increasing concentrations of Ac-YVAD-cmk and Z-VAD-fmk together with nigericin (Figure S1). To examine the dependency of potassium efflux, Pam_3_CSK_4_-primed THP-1 macrophages were stimulated in the presence of potassium chloride (75 mM, 6781.3, Carl Roth, Karlsruhe, Germany). To determine the contribution of the NLRP3 inflammasome, THP1 KO NLRP3 macrophages were primed with 1 μg/ml Pam_3_CSK_4_ for 3 h followed by stimulation with BzATP, ATP or nigericin for 3 h.

### ELISA

After 3 h of stimulation of Pam_3_CSK_4_-primed PBMCs or THP-1 macrophages or after 6 h of stimulation of Pam_3_CSK_4_-primed THP-1 monocytes cell culture supernatants were collected and analyzed for IL-1β release using commercially available ELISA kits (88–7261-88 from Thermofisher Scientific, Darmstadt, Germany).

### LDH

LDH assay was performed according to the manufacturer’s instructions (Thermofisher Scientific, Darmstadt, Germany). The percentage of LDH release was calculated compared to 100% cell lysis control.

### RNA isolation, cDNA synthesis and qRT-PCR

Total RNA isolation was performed using innuPREP RNA Mini Kit 2.0 (845-KS-2040050, AnalytikJena, Jena,Germany) according to the manufacturer’s protocol. Hence, cDNA was synthesized with the help of iScript cDNA Synthesis Kit (1708891, Bio-Rad, Feldkirchen, Germany). Quantitative real-time RT-PCR (qRT-PCR) was performed as previously described [20, 21]. Primers (synthesized by TIB Molbiol, Berlin, Germany or eurofins genomics, Ebersberg, Germany) with the following sequences were used: GAPDH, 5’-CTCTCTGCTCCTCCTGTTCGAC-3’ and 5’-TGAGCGATGTGGCTCGGCT-3’; IL1B, 5’-TGGAGCAACAAGTGGTGT-3’ and 5’-TTGGGATCTACACTCTCCAGC-3’; IL18, 5´-TGCCAACTCTGGCTGCTAAA-3´ and 5´-TTGTTGCGAGAGAAGCGAT-3´, PRTN3, 5´- GCCGGCCACATAACATTTGC-3´and 5´-TACCCGCGTGAAGAAGTCAGG-3´; ELANE, 5´-AACGGCTACGACCCCGTAAA-3´ and 5´-CTGCACGTTGGCGTTGATGG-3´; CTSG, 5´-GCTGAGGCAGGGGAGATCATCG-3´ and 5´-GGGTGTTTTCCCGTCTCTGGA-3´

Fold difference in gene expression was normalized to the housekeeping gene GAPDH showing the most constant expression levels. The reaction mix containing cDNA template, primers and SYBR Green (iTaq Universal SYBR Green Supermix; 172–5125, Bio-Rad) was run under the conditions as previously described.

### Statistical analysis

Data are expressed as means + SEM. For multiple comparisons, statistically significant differences were determined by one-way ANOVA followed by a Dunnett´s or Tukey´s post-test and considered significant at *P ≤ 0.05, **P ≤ 0.01, ***P ≤ 0.001, ****P ≤ 0.0001. For studies of inhibitory effects ATP, BzATP or nigericin induced IL-1β release was set to 100%. All other values were calculated accordingly. Statistical differences were assessed by one-sample t test against 100%. Statistical analysis was performed using GraphPad Prism software.

## Results

### Pam_3_CSK_4_-primed human THP-1 macrophages are a suitable model for studying NLRP3-mediated IL-1β release

**Fig. 1:**
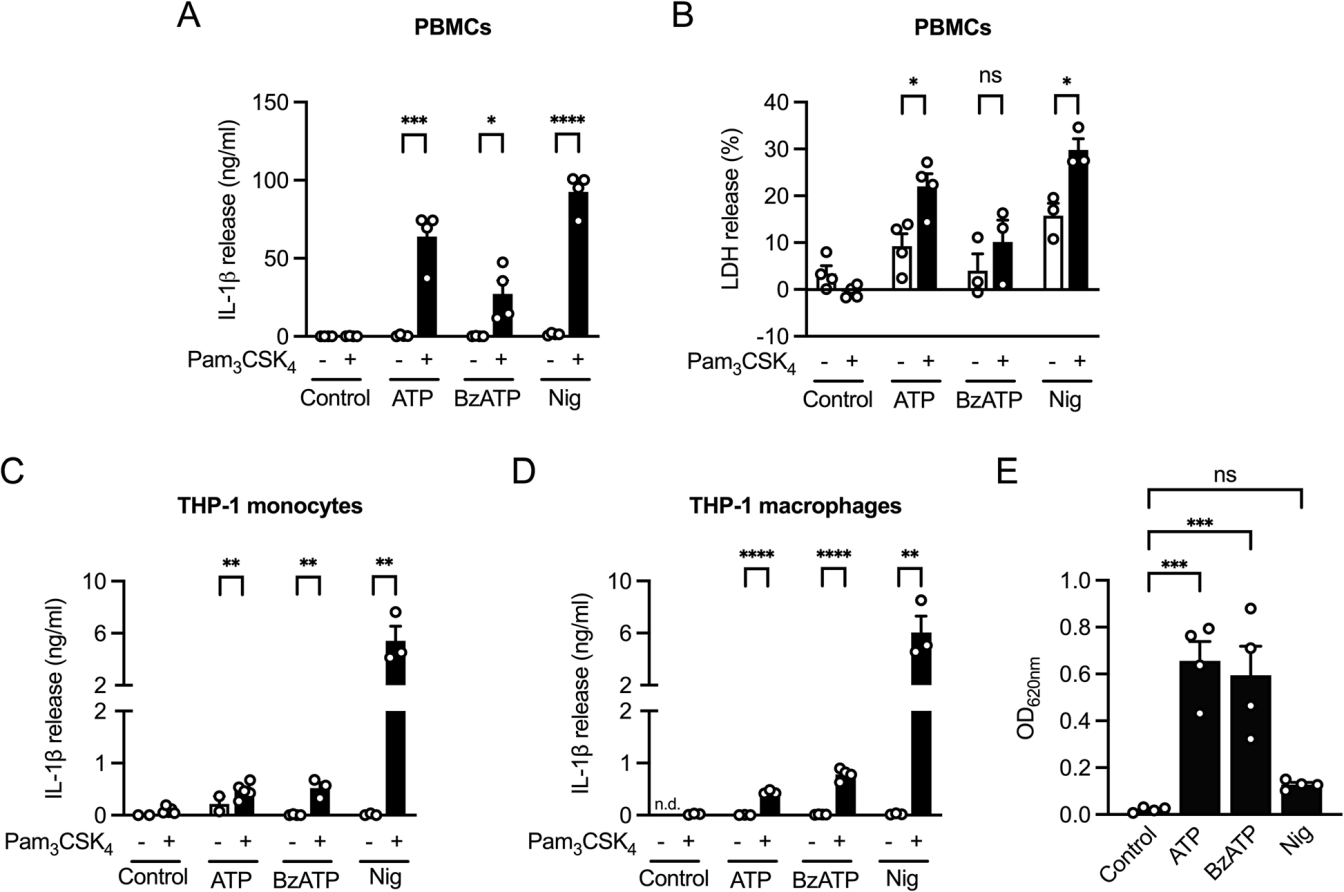
Priming of macrophages is essential to induce release of bioactive IL-1β. **A** and **B** PBMCs were primed with Pam_3_CSK_4_ and then stimulated with ATP, BzATP or nigericin for 3 h. Supernatants were analyzed (**A**) for IL-1β concentration by ELISA and (**B**) LDH-release. Results are expressed as % of maximal LDH-release. Mean + SEM (*n* = 3—4). **C** THP-1 monocytes were primed with Pam_3_CSK_4_ and afterwards stimulated with ATP, BzATP and nigericin for 6 h. IL-1β concentration in the supernatants was analyzed by ELISA. Mean + SEM (*n* = 3—5). **D** THP-1 macrophages were primed and stimulated as described in (**A**). IL-1β concentration in the supernatants was analyzed by ELISA. Mean + SEM (*n* = 3—4). Two-tailed two-sample t test, ns ≥ 0.05, *P ≤ 0.05, **P ≤ 0.01, ***P ≤ 0.001, ****P ≤ 0.0001. **E** THP-1 macrophages were primed with Pam_3_CSK_4_. Cells were then stimulated with ATP, BzATP and nigericin for 3 h. Supernatants from stimulated THP-1 macrophages were transferred on HEK-blue IL-1β reporter cells. SEAP production was detected by QUANTI-Blue and optical density was measured at 620 nm. Mean + SEM (*n* = 4). One-way ANOVA followed by Dunnett´s post-test, ns ≥ 0.05, *P ≤ 0.05, **P ≤ 0.01, ***P ≤ 0.001, ****P ≤ 0.0001

We primed peripheral blood mononuclear cells (PBMCs) with the TLR2/1 ligand Pam_3_CSK_4_ followed by stimulation with ATP, BzATP, and nigericin. We confirmed that priming was necessary to induce IL-1β release in PBMCs (Fig. 1A). To determine pyroptotic cell death, release of lactate dehydrogenase (LDH) was quantified [22]. ATP, BzATP and nigericin increased LDH release in Pam_3_CSK_4_ primed PBMCs by 22%, 7%, and 25%, respectively, compared to unstimulated PBMCs (Fig. 1B). The human THP-1 cell line is a well-established and widely used model for studying activation of the NLRP3 inflammasome [23]. To confirm this, we stimulated both THP-1 monocytes and THP-1 macrophages identically to PBMCs (Fig. 1C, and D). We observed a strong increase in IL-1β release after stimulation with ATP, BzATP or nigericin, although the response was less than that observed in PBMCs.

To investigate the bioactivity of the secreted IL-1β, we added the supernatants of stimulated THP-1 macrophages to HEK-blue IL-1β reporter cells (Fig. 1E). Bioactive IL-1β stimulates the IL-1 receptor of HEK-blue cells, which is accompanied by an NF-κB-mediated SEAP release, reflected by a colorimetric shift and higher absorbance of detection media. Supernatants of Pam_3_CSK_4_-primed THP-1 macrophages stimulated with ATP and BzATP resulted in a significant increase in absorbance confirming that ATP and BzATP induced the bioactive form of IL-1β. Although nigericin strongly induced IL-1β release in THP-1 macrophages we did not observe a significant increase in absorbance compared to control, which might be explained by cytotoxic effects of remaining nigericin in the supernatant.

### P2X7 receptor induced IL-1β secretion is mostly NLRP3 independent

**Fig. 2:**
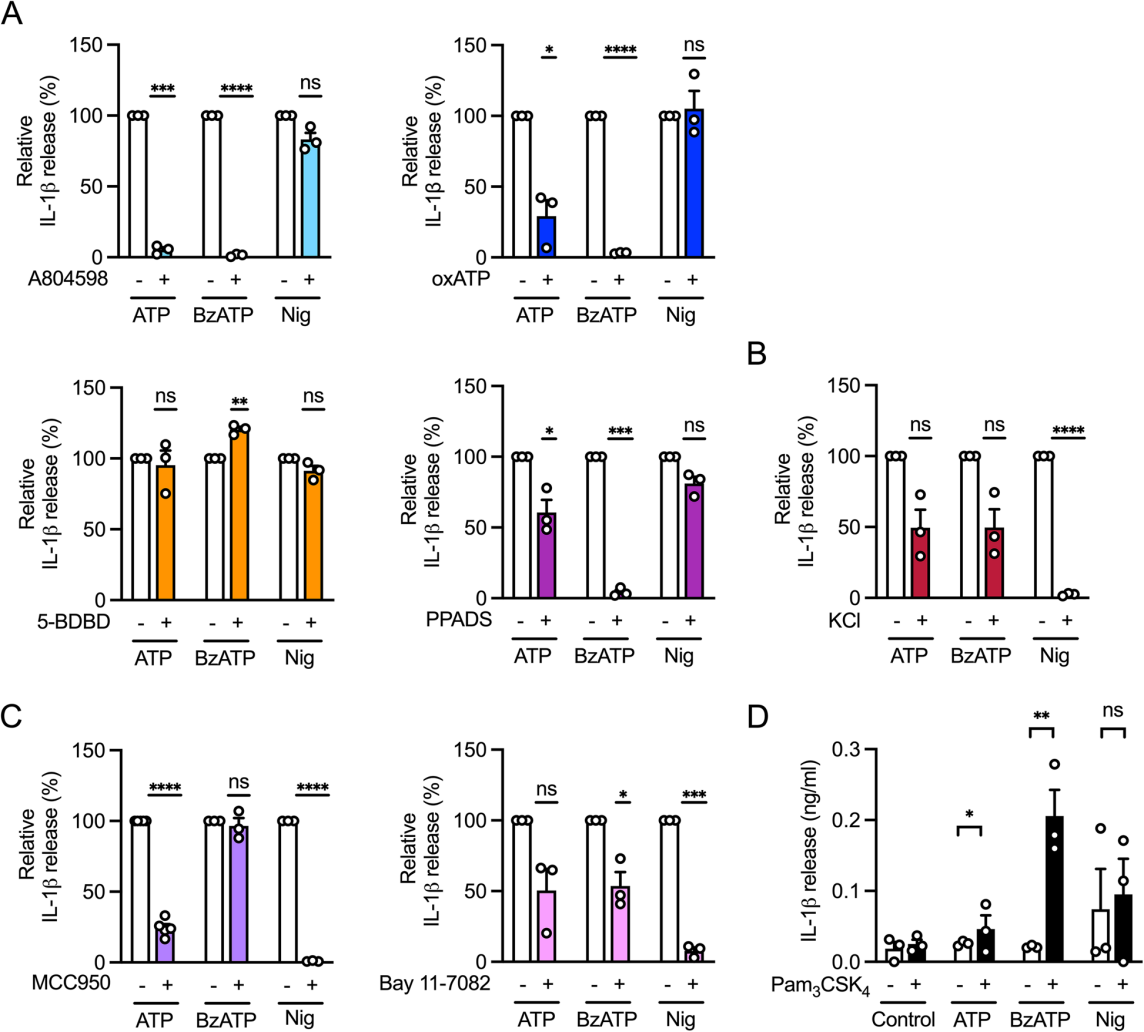
P2X7-mediated IL-1β release is NLRP3 independent. **A** THP-1 macrophages were primed with Pam_3_CSK_4_ and then stimulated with ATP, BzATP or nigericin for 3 h. Antagonists of P2X7 receptor (A-804598, oxATP), P2X4 receptor (5-BDBD) or P2X receptors (PPADS) were added 1 h before stimulation. IL-1β concentration in the supernatants was analyzed by ELISA. ATP, BzATP or nigericin induced IL-1β release was set to 100%. Mean + SEM (*n* = 3). One-sample t test against 100%, ns ≥ 0.05, *P ≤ 0.05, **P ≤ 0.01, ***P ≤ 0.001, ****P ≤ 0.0001. **B** THP-1 macrophages were primed with Pam_3_CSK_4_. Potassium chloride was added together with ATP, BzATP or nigericin for 3 h. IL-1β concentration in the supernatants was analyzed by ELISA. ATP, BzATP or nigericin induced IL-1β release was set to 100%. Mean + SEM (*n* = 3). One-sample t test against 100%, *P ≤ 0.05, ****P ≤ 0.0001. **C** THP-1 macrophages were primed with Pam_3_CSK_4_ and then stimulated with ATP, BzATP or nigericin for 3 h. NLRP3 inhibitors MCC950 or Bay 11–7082 were added 1 h before stimulation. IL-1β concentration in the supernatants was analyzed by ELISA. ATP, BzATP or nigericin induced IL-1β release was set to 100%. Mean + SEM (*n* = 3—4). One-sample t test against 100%, ns ≥ 0.05, *P ≤ 0.05, ***P ≤ 0.001, ****P ≤ 0.0001 (**D**) NLRP3 KO THP-1 macrophages were primed with Pam_3_CSK_4_. Cells were then stimulated with ATP, BzATP or nigericin for 3 h. IL-1β concentration in the supernatants was analyzed by ELISA. Mean + SEM (*n* = 3). Two-tailed two sample t test, ns ≥ 0.05, *P ≤ 0.05, **P ≤ 0.01, ***P ≤ 0.001, ****P ≤ 0.0001

To determine whether ATP mediates the release of IL-1β via the P2X7 receptor or whether other receptors are also involved, we tested various P2X receptor antagonists. BzATP, a more potent P2X7 receptor agonist [18, 19], served as a reference. In THP-1 macrophages, IL-1β release by ATP and BzATP was inhibited in the presence of the P2X7 receptor-selective antagonists A804598 and oxATP (Fig. 2A). The inhibition of ATP-induced cytokine release by the irreversible antagonist oxATP was less pronounced than by the non-competitive reversible antagonist A804598 [24, 25]. Addition of the P2X4 receptor-specific antagonist 5-BDBD [26] did not decrease IL-1β release by ATP and BzATP. PPADS, a selective purinergic P2X receptor antagonist [27, 28], reduced the release of IL-1β by THP-1 macrophages stimulated with ATP and BzATP to 60.6% and 4.2%, respectively. As expected, IL-1β release mediated by the potassium ionophore nigericin was not affected by the addition of P2X receptor antagonists.

It has been proposed that whether the P2X7 receptor is activated by ATP or BzATP, or nigericin is used as an ionophore, ultimately K^+^ efflux from the intracellular space is critical for activation of the NLRP3 inflammasome [11, 12]. To determine the optimal extracellular potassium chloride concentration required to inhibit IL-1β secretion, we incubated increasing potassium concentrations together with nigericin (Figure S2A). A concentration of 75 mM extracellular potassium chloride proved to be nontoxic (Figure S2B). Nigericin-induced IL-1β release by THP-1 macrophages was completely blocked by 75 mM extracellular potassium chloride, whereas IL-1β secretion induced by ATP or BzATP could be reduced only by 50% (Fig. 2B). Assuming that the release of IL-1β is dependent on NLRP3, we tested the specific NLRP3 inflammasome inhibitor MCC950 [29]. MCC950 suppressed IL-1β secretion induced by ATP and nigericin to 24.4% and 0.9% (Fig. 2C). IL-1β release induced by BzATP was not affected, suggesting an NLRP3-independent release of IL-1β. We attempted to verify this result by using the NLRP3 inflammasome inhibitor Bay 11–7082 [30]. In the presence of Bay 11–7082, ATP-, BzATP-, and nigericin-induced IL-1β release was reduced to 50.5%, 53.7%, and 7.8%, respectively (Fig. 2C). The observed differences between MCC950 and the non-specific NLRP3 inhibitor Bay 11–7082 could be explained by interference of Bay 11–7082 with NF-κB signaling [7], therefore being able to reduce IL-1β release beyond direct inhibition of the NLRP3 inflammasome. To further support our results independently of pharmacological inhibition, we used THP-1 macrophages that are deficient for NLRP3. Stimulation with BzATP or ATP following priming resulted in a significant increase in IL-1β release, with IL-1β release being higher by BzATP than ATP. In contrast, nigericin failed to increase IL-1β release in primed NLRP3 knockout THP-1 macrophages (Fig. 2D).

### P2X7 receptor induced IL-1β secretion is partially caspase independent

**Fig. 3:**
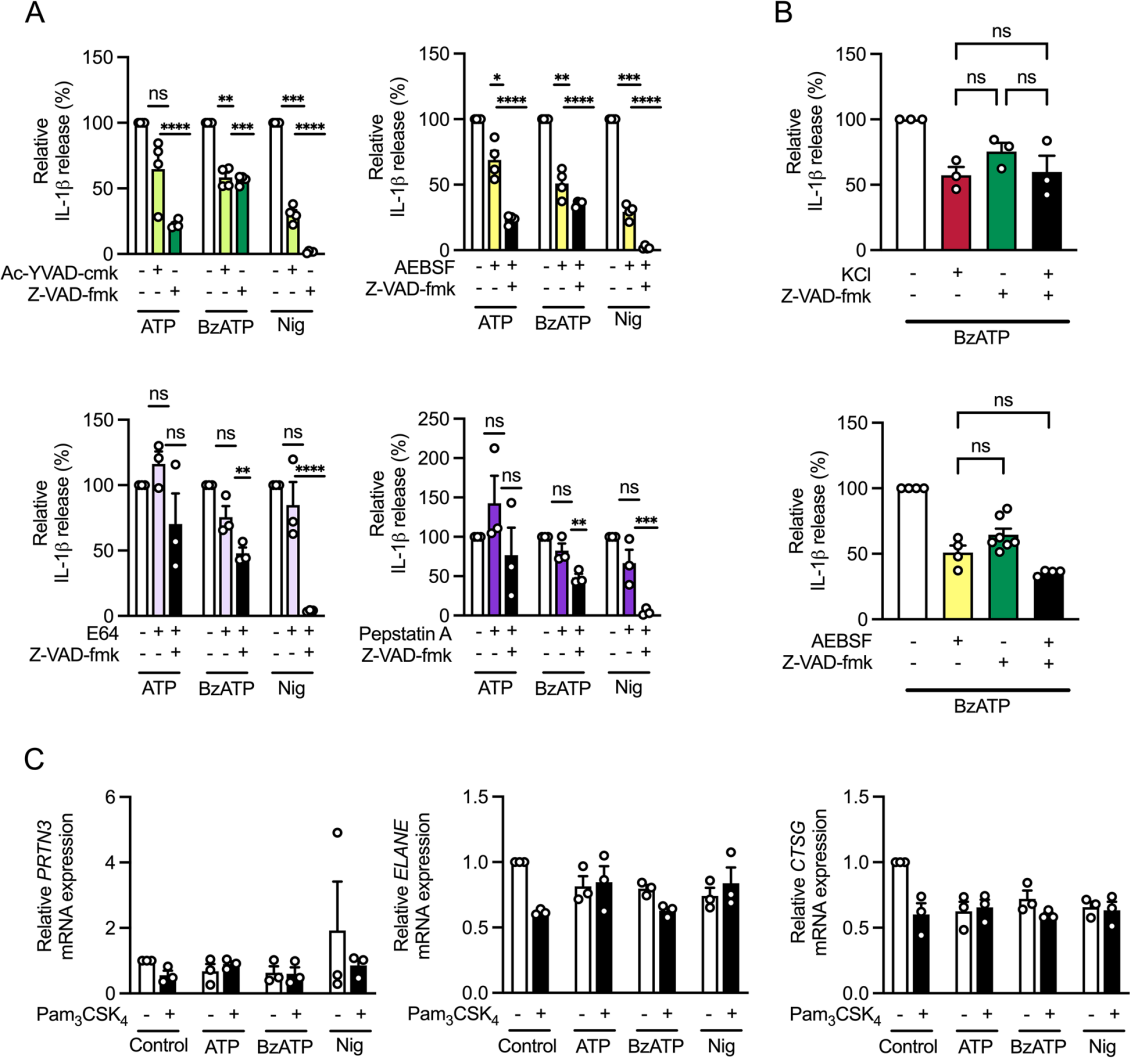
Influence of caspases, serine proteases, cysteine proteases and aspartic proteases on IL-1β release. **A** THP-1 macrophages were primed with Pam_3_CSK_4_ and then stimulated with ATP, BzATP or nigericin for 3 h. Inhibitors of Caspase-1 (Ac-YVAD-cmk) or pan-caspase (Z-VAD-fmk), serine protease (AEBSF), cysteine protease (E64), aspartic protease (pepstatin A) were added 1 h before stimulation. IL-1β concentration in the supernatants was analyzed by ELISA. ATP, BzATP or nigericin induced IL-1β release was set to 100%. Mean + SEM (*n* = 3—4). One-sample t test against 100%, ns ≥ 0.05, *P ≤ 0.05, **P ≤ 0.01, ***P ≤ 0.001, ****P ≤ 0.0001. **B** THP-1 macrophages were primed with Pam_3_CSK_4_ and then stimulated with BzATP in the absence or presence of KCl for 3 h. Pan-caspase inhibitor (Z-VAD-fmk) or serine protease inhibitor (AEBSF) was added 1 h before stimulation. IL-1β concentration in the supernatants was analyzed by ELISA. BzATP induced IL-1β release was set to 100%. Mean + SEM (*n* = 3—7). One-way ANOVA followed by Tukey´s post-test, ns ≥ 0.05 (**C**) THP-1 macrophages were primed with Pam_3_CSK_4_ and then stimulated with ATP, BzATP or nigericin for 3 h. Gene expression of *PRTN3* and *ELANE* and *CTSG* was normalized to *GAPDH*. Mean + SEM (*n* = 3)

NLRP3 oligomerization leads to self-cleavage of pro-caspase-1 into its active form [31]. Subsequently, active caspase-1 cleaves pro-IL-1β into mature and bioactive IL-1β. To determine to which extent IL-1β secretion depends on caspases, we used the caspase-1 inhibitor Ac-YVAD-cmk and the pan-caspase inhibitor Z-VAD-fmk. Inhibition of caspase-1 activity decreased IL-1β secretion induced by ATP, BzATP and nigericin to 64.8%, 58.4% and 30.3%, respectively, without having toxic effects (Fig. 3A, and S3). The effect of the pan-caspase inhibitor Z-VAD-fmk on IL-1β release mediated by ATP and nigericin was even more pronounced and reduced cytokine levels to 22.6% and 1.6%, respectively. However, BzATP-induced IL-1β release was reduced to 56.4% and showed comparable levels to those observed for the specific caspase-1 inhibitor.

To elucidate the involvement of other proteases in IL-1β secretion, we used inhibitors of serine-, cysteine-, and aspartic proteases. In the presence of the serine protease inhibitor AEBSF we observed a decrease in IL-1β secretion mediated by ATP, BzATP and nigericin (Fig. 3A). When AEBSF was used in combination with a pan-caspase inhibitor, IL-1β secretion by ATP and BzATP was further reduced, whereas IL-1β release induced by nigericin was completely blocked. IL-1β levels secreted by ATP-, BzATP- and nigericin-stimulated macrophages remained unchanged in the presence of the cysteine protease inhibitor E64 or the aspartic protease inhibitor pepstatin A. In contrast, the combination of E64 or pepstatin A with Z-VAD-fmk reduced IL-1β release mediated by ATP, BzATP, and nigericin. No toxic effects were observed (Figure S3). To determine whether caspase-dependent IL-1β release by BzATP was related to potassium efflux or whether these signaling cascades occur separately, we inhibited potassium efflux and caspases simultaneously. A synergistic effect could be ruled out, as the combination of potassium chloride and pan-caspase inhibitor Z-VAD-fmk reduced BzATP-induced IL-1β release to levels similar to the addition of potassium chloride alone (Fig. 3B). This trend was repeated when using the serine protease inhibitor AEBSF (Fig. 3B). Combining AEBSF with Z-VAD-fmk could not reduce BzATP-mediated IL-1β release any further than AEBSF alone. This observation suggests that potassium efflux, serine proteases, and caspases operate in the same signaling pathway. Gene expression levels of the serine proteases proteinase 3 (*PRTN3*), neutrophil elastase (*ELANE*), or cathepsin G (*CTSG*), which have been shown to alternatively process IL-1β compared to caspase-1 [32], were not affected by stimulation with ATP, BzATP or nigericin (Fig. 3C).

### Priming with different TLR ligands affects signal transduction of P2X7 mediated IL-1β release

**Fig. 4:**
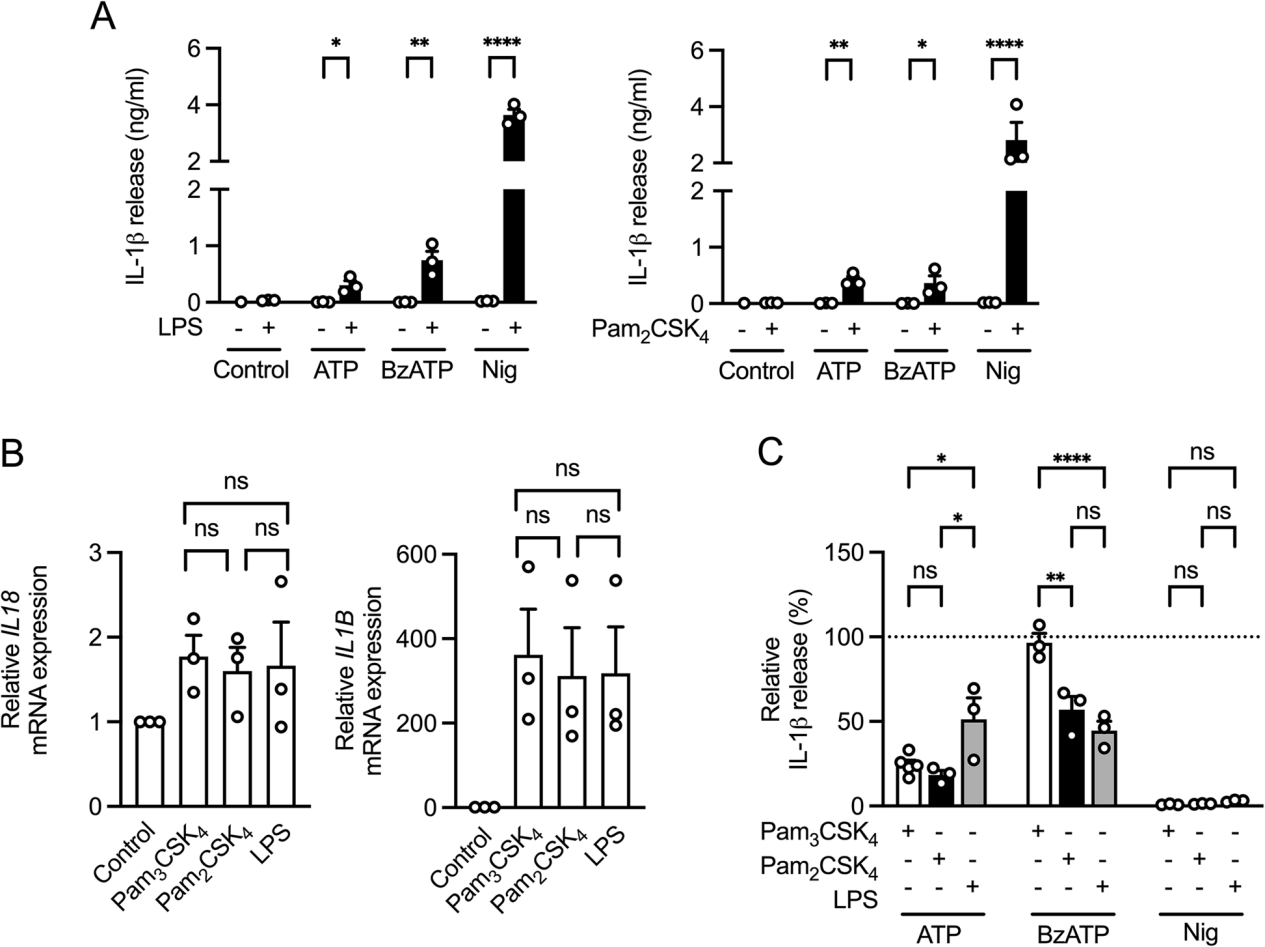
Influence of priming with different TLR ligands on IL-1β release. **A** THP-1 macrophages were primed with LPS or Pam_2_CSK_4_. Cells were then stimulated with ATP, BzATP or nigericin for 3 h. IL-1β concentration in the supernatants was analyzed by ELISA. Mean + SEM (*n* = 3). Two-tailed two-sample t test, ns ≥ 0.05, *P ≤ 0.05, **P ≤ 0.01, ***P ≤ 0.001, ****P ≤ 0.0001. **B** THP-1 macrophages were primed with Pam_3_CSK_4_, Pam_2_CSK_4_ or LPS. Gene expression of IL18 or IL1B was normalized to GAPDH. Mean + SEM (*n* = 3). One-way ANOVA followed by Tukey´s post-test, ns ≥ 0.05. **C** THP-1 macrophages were primed with Pam_3_CSK_4_, Pam_2_CSK_4_ or LPS and then stimulated with ATP, BzATP or nigericin. NLRP3 inhibitor (MCC950) was added 1 h before stimulation. IL-1β concentration in the supernatants was analyzed by ELISA. ATP, BzATP or nigericin induced IL-1β release was set to 100%. Mean + SEM (*n* = 3—5). One-way ANOVA followed by Tukey´s post-test, ns ≥ 0.05, *P ≤ 0.05, **P ≤ 0.01

Not only TLR2/1 ligands can serve as priming signal for NLRP3 inflammasome activation. Therefore, we also used the TLR2/6 ligand Pam_2_CSK_4_ and the TLR4 ligand LPS and observed increased IL-1β release after stimulation with ATP, BzATP, or nigericin (Fig. 4A). After demonstrating that priming is essential to induce IL-1β release in THP-1 macrophages, we wanted to further characterize the role of different TLR signaling pathways. The relative gene expressions of *IL1B* and *IL18* did not show a significant difference between TLR2 and TLR4 signaling (Fig. 4B). In macrophages stimulated with nigericin, inhibition of the NLRP3 inflammasome resulted in a comparable decrease in IL-1β release, regardless of which TLR ligand was used for priming (Fig. 4C). Furthermore, ATP-induced IL-1β release consistently showed partial independence from NLRP3 regardless of the TLR ligand used for priming. However, the extent of NLRP3 independence was different. Although incubation with MCC950 did not result in significant differences in IL-1β release when priming with TLR2/1 or TLR2/6 agonists, priming with a TLR4 agonist led to a significantly lower level of inhibition. Opposite results were observed in BzATP-stimulated cells. As demonstrated above (Fig. 2C), cytokine levels induced by BzATP remained unchanged in the presence of the NLRP3 inflammasome inhibitor MCC950 when priming was performed by TLR2/1 ligand Pam_3_CSK_4_. In contrast, inhibition of NLRP3 reduced BzATP-induced IL-1β cytokine levels to 44.6% and 57.0% in LPS- and Pam_2_CSK_4_-primed cells, respectively, indicating that TLR signaling differentially modulates signal transduction of P2X7-mediated IL-1β release (Fig. 4C).

## Discussion

Although our findings support the importance of the NLRP3 inflammasome in P2X7 receptor-mediated IL-1β release [17], we propose an NLRP3-independent pathway in human macrophages. This previously unrecognized mechanism underlines the value of inhibiting the P2X7 receptor in inflammatory diseases [33–35] and highlights species-specific differences for IL-1β secretion between mouse and human macrophages. We demonstrated that stimulation with the P2X7 receptor ligand ATP led to NLRP3-independent IL-1β release. In Pam_3_CSK_4_-primed human THP-1 macrophages lacking NLRP3, stimulation with ATP led to a significant increase in IL-1β release. These effects were even more pronounced for BzATP, a synthetic ATP derivative and a more potent P2X7 receptor ligand [18].

A limitation of the present study is the use of the THP-1 cell line, which may deviate from primary cells. THP-1 cells are widely used to study the NLRP3 inflammasome [36], however, future studies should include human primary macrophages to confirm our findings. Yet, our results are in line with previous studies showing that the NLRP3 inhibitor MCC950 only partially inhibits IL-1β release in primary human macrophages, while the responses are completely dependent on NLRP3 in mouse macrophages [29, 37, 38].

We confirmed full NLRP3-dependency of nigericin induced IL-1β release [11], however, the contribution of NLRP3 was dispensable for ATP and BzATP. Thus, we aimed to investigate the role of the P2X7 receptor in IL-1β secretion, although it is believed to do so in a NLRP3-dependent manner [17]. Inhibitors of P2X and selective inhibitors of P2X7 receptors blocked BzATP but not nigericin induced IL-1β release indicating that NLRP3-independent IL-1β secretion is fully attributable to the P2X7 receptor. This finding is supported by results obtained with the selective P2X7 inhibitor A438079 in LPS-primed mouse macrophages [39]. Although we did not include P2X7 knockout cells in our study, we focused on various pharmacological inhibitors of P2X receptors with different selectivity and mode of action.

High extracellular potassium concentrations inhibit NLRP3 activation and IL-1β release [11, 12]. Our results suggest that P2X7 receptor-mediated IL-1β release is only partially dependent on K^+^ efflux, which differs from the previous concept established in mouse macrophages [11]. Our findings are consistent with results obtained in human macrophages [37], although a link to the P2X7 receptor and NLRP3 independence was not established previously. Our findings do not refute potassium efflux being the common trigger of NLRP3 inflammasome activation [11], but further underline the existence of a P2X7 receptor-facilitated NLRP3-independent IL-1β releasing pathway.

Caspase-1 is a central player in inflammasome-induced IL-1β release [5]. Although other caspases, such as caspase-8, can process IL-1β [40], only caspase-1 is involved in BzATP-induced IL-1β release. ATP and nigericin activate additional caspases, as pan-caspase inhibition further inhibited IL-1β release. While nigericin-induced IL-1β release is fully caspase-dependent, the P2X7 receptor agonists ATP and BzATP trigger IL-1β release partially caspase independent. This contradicts the notion that P2X7 receptor activation leads to IL-1β release via caspase-1 only [37, 41, 42], and once again emphasizes the differences of important signaling pathways between human and mouse macrophages. Furthermore, our results prove that K^+^ efflux is associated with caspase-1 for P2X7 receptor-dependent IL-1β release.

Caspase and NLRP3 independent mechanisms for IL-1β processing have been described, including serine proteases that are involved in caspase-1-independent IL-1β release [43, 44]. A significant reduction of IL-1β release was achieved in response to all stimuli tested by inhibition of serine protease. Serine protease inhibition reduces IL-1β release by human macrophages under acidic stress [37]. We show that serine proteases are also involved in both the NLRP3-dependent and -independent release of IL-1β. Simultaneous inhibition of caspases and serine proteases led to a similar reduction in IL-1β secretion compared to caspase inhibition alone, indicating that serin proteases are only involved in the caspase/IL-1β axis. Because a substantial level of IL-1β release remained after simultaneous inhibition of caspase and serine protease, we checked the involvement of cysteine and aspartic proteases. Aspartic protease inhibition reduced IL-1β release by human macrophages if stimulated with lactic acid [37]. We did not observe a reduction in IL-1β release with inhibition of either class of proteases.

Lastly, priming with TLR agonists differentially modulates NLRP3 dependency of P2X7 mediated IL-1β release. Priming with the TLR2/1 ligand Pam_3_CSK_4_ led to completely NLRP3-independent IL-1β release in BzATP-stimulated macrophages, while priming with TLR2/6 and TLR4 ligands led to some degree of NLRP3 dependency. Therefore, it would be interesting to determine the underlying mechanisms in the NLRP3 priming pathway triggered by TLR ligands.

## Conclusions

**Fig. 5:**
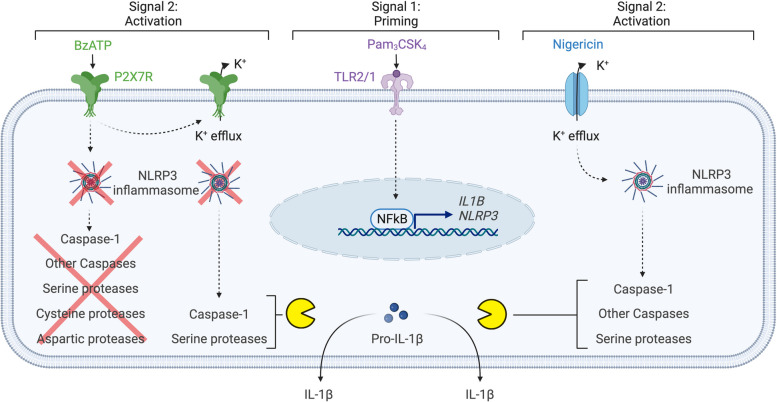
Proposed mechanism for NLRP3-independent IL-1β release by human macrophages after P2X7 receptor activation. Activation of TLR2/1 by Pam_3_CSK_4_ leads to NF-κB mediated production of pro-IL-1β and components of NLRP3 inflammasome. Nigericin-mediated K^+^ efflux leads to NLRP3 inflammasome oligomerization causing serine protease- and caspase-dependent IL-1β release. In contrast, P2X7 receptor stimulation by BzATP potentially initiates two distinct IL-1β releasing mechanisms that are both NLRP3-independent. One mechanism relies on K^+^ efflux and requires activation of caspase-1 and serine proteases. The other mechanism is independent of K^+^ efflux, caspases, and serine-, cysteine-, and aspartic proteases, suggesting the involvement of other mechanisms for proteolytic processing of IL-1β

In summary, we propose an independent pathway of NLRP3 and caspase for IL-1β release following activation of the P2X7 receptor in human macrophages (Fig. 5). In this pathway, cleavage of pro-IL-1β is not caused by caspase-1, cysteine protease, or aspartic proteases, indicating an unknown mechanism of cleavage of pro-IL-1β to its active form. Our findings have significance for understanding P2X7 receptor-mediated IL-1β release, which will facilitate the development of new therapeutic strategies aimed at modulating inflammatory responses.

### Supplementary Information

**Additional file 1:** **Supplementary figures S1-S3.**

## Data Availability

All data generated and analyzed during this study are included in the manuscript.

## Abbreviations

ATP: Adenosine 5’-triphosphate
BzATP: 2′(3′)-O-(4-benzoylbenzoyl) adenosine 5′-triphosphate
IL: Interleukin
LDH: Lactate dehydrogenase
NLRP3: Nucleotide-binding oligomerization domain-like receptor family pyrin domain-containing protein 3
TLR: Toll-like receptor
