# Supplementary material for: P2X7 receptor activation leads to NLRP3-independent IL-1β release by human macrophages
Source: Cell Commun Signal. 2023 Nov 23;21:335. doi: 10.1186/s12964-023-01356-1 (PMC10666422; doi:10.1186/s12964-023-01356-1)
Supplement: Supplementary file 2 — Additional file 1: Supplementary figures S1-S3. [file 12964_2023_1356_MOESM1_ESM.pdf]

Supplementary Information

P2X7 receptor activation leads to NLRP3-independent IL-1β release by human macrophages

Judith Bockstiegel[1†], Jonas Engelhardt[1†], Günther Weindl[1*]

[1]Pharmaceutical Institute, University of Bonn, 53121 Bonn, Germany

[†]Judith Bockstiegel and Jonas Engelhardt contributed equally to this work.

[*]Correspondence: Dr. Günther Weindl, Pharmacology and Toxicology section, Pharmaceutical Institute, University of Bonn, 53121 Bonn, Germany. Phone: +49 228 739103, e-mail: guenther.weindl@uni-bonn.de, ORCID: 0000-0002-4493-7597

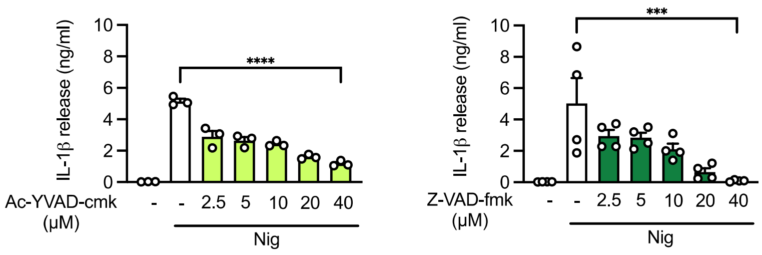

Figure S1. THP-1 macrophages were primed with Pam_3CSK_4 and then stimulated with nigericin for 3 h. Inhibitors of caspase-1 (Ac-YVAD-cmk) or pan-caspase (Z-VAD-fmk) in increasing concentrations were added 1 h before stimulation. IL-1β concentration in the supernatants was analyzed by ELISA. Mean + SEM (n = 3 - 4). One-way ANOVA followed by Dunnett´s post-test, ***P ≤ 0.001, ****P ≤ 0.0001.

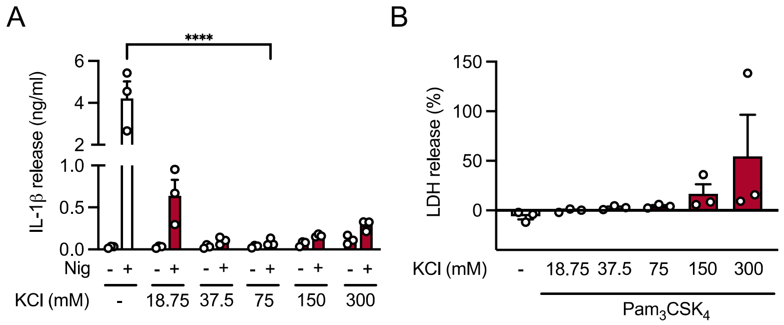

Figure S2. THP-1 macrophages were primed with Pam_3CSK_4. Potassium chloride was added in increasing concentrations (**A**) together with or (**B**) without nigericin for 3 h. (A) IL-1β concentration in the supernatants was analyzed by ELISA. Mean + SEM (n = 3). (B) LDH release in the supernatants was analyzed. Results are expressed as % of maximal LDH-release. Mean + SEM (n = 3). One-way ANOVA followed by Dunnett´s post-test, ****P ≤ 0.0001.

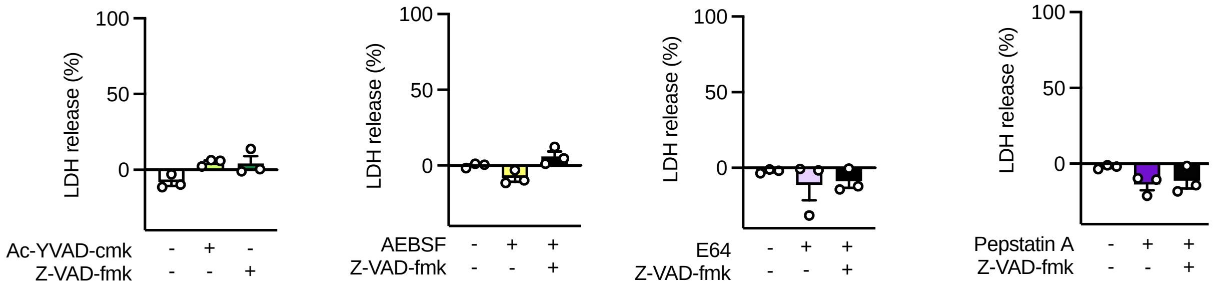

Figure S3. THP-1 macrophages were primed with Pam_3CSK_4 and then incubated with cell culture media for 3 h. Inhibitors of caspase-1 (Ac-YVAD-cmk) or pan-caspase (Z-VAD-fmk), serine protease (AEBSF), cysteine protease (E64), aspartic protease (pepstatin A) were added 1 h before incubation. LDH release in the supernatants was analyzed. Results are expressed as % of maximal LDH-release. Mean + SEM (n = 3).
